# Phylogenomics of MADS-Box Genes in Plants — Two Opposing Life Styles in One Gene Family

**DOI:** 10.3390/biology2031150

**Published:** 2013-09-12

**Authors:** Lydia Gramzow, Günter Theißen

**Affiliations:** Department of Genetics, Friedrich Schiller University Jena, Philosophenweg 12, 07743 Jena, Germany; E-Mail: guenter.theissen@uni-jena.de

**Keywords:** gene duplication, gene family evolution, gene expansion, transcription factor, whole genome duplication

## Abstract

The development of multicellular eukaryotes, according to their body plan, is often directed by members of multigene families that encode transcription factors. MADS (for MINICHROMOSOME MAINTENANCE1, AGAMOUS, DEFICIENS and SERUM RESPONSE FACTOR)-box genes form one of those families controlling nearly all major aspects of plant development. Knowing the complete complement of MADS-box genes in sequenced plant genomes will allow a better understanding of the evolutionary patterns of these genes and the association of their evolution with the evolution of plant morphologies. Here, we have applied a combination of automatic and manual annotations to identify the complete set of MADS-box genes in 17 plant genomes. Furthermore, three plant genomes were reanalyzed and published datasets were used for four genomes such that more than 2,600 genes from 24 species were classified into the two types of MADS-box genes, Type I and Type II. Our results extend previous studies, highlighting the remarkably different evolutionary patterns of Type I and Type II genes and provide a basis for further studies on the evolution and function of MADS-box genes.

## 1. Introduction

MADS-box genes encode transcription factors and have been identified in nearly all groups of eukaryotes [1]. They are major regulators of development [2,3]. The acronym MADS is derived from the four founding members of this gene family: *MINICHROMOSOME MAINTENANCE1* from *Saccharomyces cerevisiae*, *AGAMOUS* from *Arabidopsis thaliana*, *DEFICIENS* from *Antirrhinum majus*, and *SERUM RESPONSE FACTOR* from *Homo sapiens* [4]. Whereas only two to six MADS-box genes are present in the genomes of animals and fungi, the number of these genes has greatly expanded in plants [2,5,6,7]. Roughly, about 100 MADS-box genes are typically found in flowering plant genomes [8,9]. The expansion and diversification of MADS-box genes in plants is closely linked to the evolution of novel structures, such as seeds, flowers, and fruits [5]. Consequently, MADS-box genes are key objects in studies of evolutionary developmental genetics in plants. Knowledge of the full complement of MADS-box genes along the plant lineage will enable a more profound comprehension of the evolution of the major land plant groups and the structures which define them.

MADS-box genes encode MADS-domain proteins. These are characterized by the highly conserved DNA-binding MADS domain which has a length of 56–60 amino acids [4]. Two types of MADS-box genes are distinguished throughout the eukaryotes, Type I and Type II [1,10]. Type II MADS-box genes of plants are also termed MIKC-type genes, referring to the typical domain structure of the encoded proteins. In MIKC-domain proteins, the MADS (M) domain is followed by an Intervening (I), a Keratin-like (K), and a C-terminal (C) domain [11]. The K domain is the second most conserved domain after the MADS domain. The two types of MADS-box genes are further subdivided into the groups Mα, Mβ, and Mγ (Type I), and MIKC^C^ and MIKC* (Type II) based on phylogenetic and structural features [9,11]. More than a dozen ancient clades of MIKC^C^-group genes are distinguished by different motifs in their C-terminal regions [2].

While only a few Type I MADS-box genes have been functionally characterized [12,13,14,15], the Type II, especially the MIKC^C^-group MADS-box genes have been well studied (e.g., [4,16,17,18,19]). In flowering plants, their functions range from root development via floral organ specification to fruit development. Interestingly, genes with similar functions and similar expression patterns in different species are generally closely related and belong to the same clade of MADS-box genes [2,5,20]. Hence, identification and determination of the phylogenetic position of MADS-box genes in plant species allows the formulation of hypotheses about both, the morphological development of these species and the function of respective genes.

**Figure 1:**
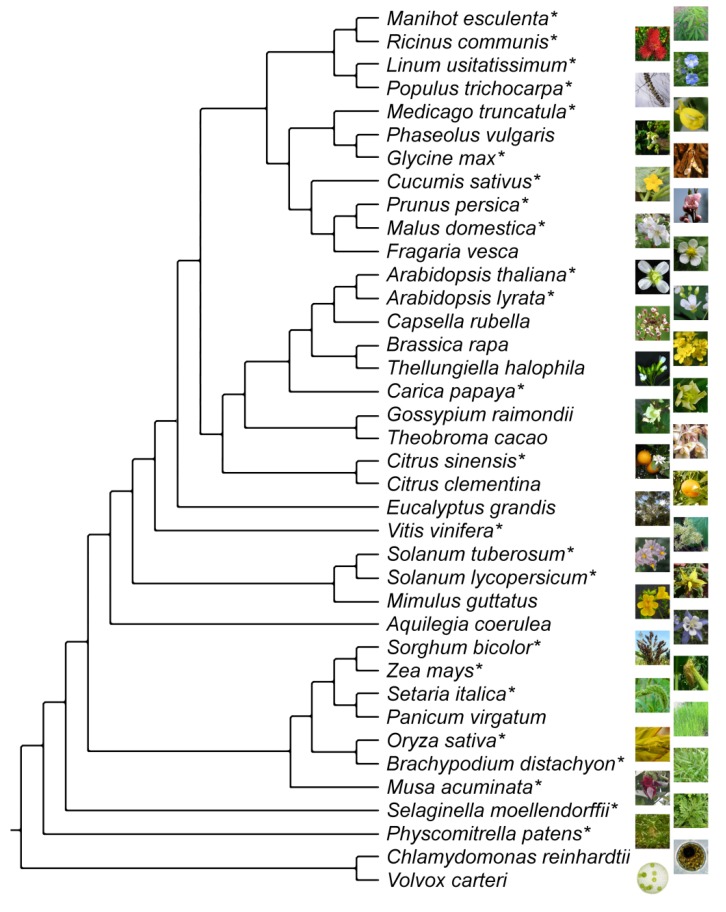
Phylogeny of land plants for which whole-genome information is now available. *Chlamydomonas reinhardtii* and *Volvox carteri* are green algae and used as representatives of the outgroup. Species for which MADS-box genes are studied here are indicated by an asterisk. The topology of the tree was taken from [29].

The complete set of MADS-box genes has been studied for the plants *Arabidopsis thaliana*, *Glycine max*, *Cucumis sativus*, *Oryza sativa*, *Populus trichocarpa*, *Selaginella moellendorffii*, and *Physcomitrella patens* [6,8,9,21,22,23,24]. Type II MADS-box genes have also been studied in *Vitis vinifera* [25]. As of June 2013, Phytozome offers access to the genomes of 28 additional land plant species (Figure 1). Many more whole-genome sequencing projects are ongoing.

Here, we use available whole-genome information to study the evolution of MADS-box genes in land plants. We have developed a pipeline which enables the fast annotation of MADS-box genes. Our pipeline first gathers MADS-box genes from gene prediction sets provided by the genome-sequencing projects. It then uses Hidden Markov Model (HMM) searches [26] of the whole genomes and the gene prediction programs GlimmerHMM [27] and FgenesH to identify MADS-box genes that have escaped the automatic annotation. Using our pipeline, we have newly identified 2,060 MADS-box genes in 17 plant species. Reanalyzing three, and using published datasets for four plant species, we analyzed a total of 2,603 MADS-box genes from 24 plant species. Approximately equal numbers of these genes were classified as Type I and Type II genes, respectively. Our phylogenies emphasize differences in the evolution of Type I and Type II genes and hence corroborate previous findings [28]. Our results will facilitate more in-depth studies on the mechanisms leading to these differences in evolutionary rates of the two types of MADS-box genes in flowering plants. 

## 2. Experimental Section

### 2.1. Sequence Data

MADS-box genes were analyzed in 24 plant genomes. Of these, MADS-box genes were newly predicted in 17 genomes, reanalyzed in three genomes (*G.max*, *C. sativus*, and *V. vinifera*) and taken from the literature for four genomes (*A. thaliana*, *O. sativa*, *S. moellendorffii*, and *P. patens*). To do so, whole-genome assemblies of all but the last four mentioned plant species were downloaded from the genome websites indicated in Table 1 or from Phytozome [29]. The coding sequences (CDS) for these species were retrieved from Phytozome.

**Table 1:** List of some species and websites from which whole-genome data were retrieved.

Species	Website
*Ricinus communis*	http://castorbean.jcvi.org/
*Medicago truncatula*	http://medicagohapmap.org/
*Carica papaya*	http://asgpb.mhpcc.hawaii.edu/papaya/
*Sorghum bicolor*	http://genome.jgi-psf.org/Sorbi1/
*Brachypodium distachyon*	http://www.brachypodium.org/

### 2.2. Annotation Pipeline

**Figure 2:**
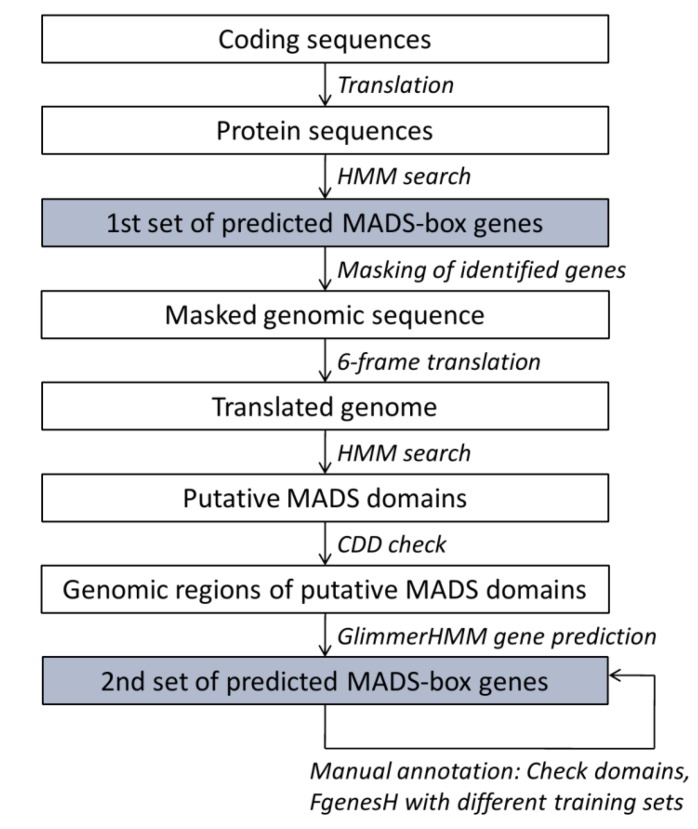
Pipeline for annotation of MADS-box genes in plants. The two sets of predicted MADS-box genes are combined to obtain what is presumably the full complement of MADS-box genes in a species.

**Figure 3:**
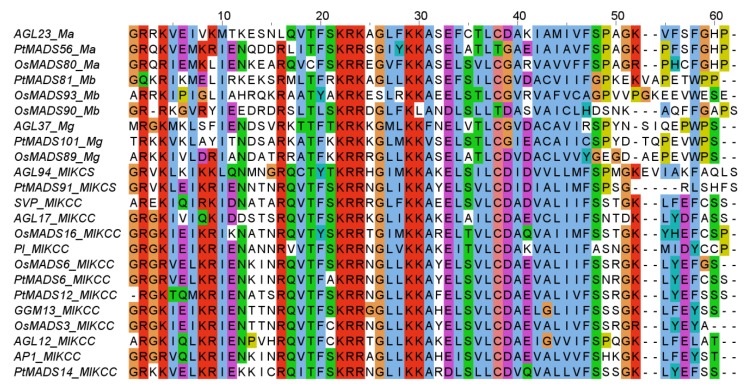
Alignment of exemplary MADS domains from all types and groups of MADS‑domain proteins from the species *Oryza sativa* (OsMADS), *Populus trichocarpa* (PtMADS), *Gnetum gnemon* (GGM), and *Arabidopsis thaliana* (all other gene names). The labelling after the underscore denotes the group: Ma, Mα; Mb, Mβ; Mg, Mγ; MIKCS, MIKC*, and MIKCC, MIKC^C^. The alignment is colored according to the Clustal X color scheme [32].

Customized perl scripts (available as Supplementary Data 1) were written to semi-automatically predict MADS-box genes in 17 plant genomes (Figure 2). Predicted CDS made available by the genome projects were translated into protein sequences and searched using the HMMer program [26] and a customized HMM of the MADS domain (Figure 3). This way, we aimed at identifying MADS‑box genes, which were already predicted by standard methods of the genome projects. The HMM is based on an alignment of exemplary MADS domains from all types and groups of MADS‑domain proteins from the species *A. thaliana*, *O. sativa*, *P. trichocarpa*, and *Gnetum gnemon* (Figure 3). The presence of the MADS domain in thereby detected sequences was confirmed by searches of the National Centre for Biotechnology Information (NCBI) conserved domains database (CDD) [30].

To detect additional MADS-box genes, which may have been missed during the automatic annotation procedure, whole-genome sequences were translated in all six reading frames. The translated genomes were again searched using the customized HMM of the MADS domain and presence of the MADS domain was counterchecked using the CDD. To annotate MADS-box genes encoding for the identified MADS domains, the corresponding genomic regions were retrieved including 300 bp upstream and 19,700 bp downstream of the start of the detected MADS box. Automatic gene predictions on these genome fragments were conducted using GlimmerHMM with the training sets of *A. thaliana* and *O. sativa* [27]. The thereby predicted genes were manually inspected and where gene predictions were short or the MADS- or K-domains were only partially included, improvements of gene predictions were attempted using FgenesH [31] with different training sets. The pipeline was tested using the genomes of *A. thaliana* and *O. sativa* where the full complements of MADS-box genes have been studied [8,9].

### 2.3. Phylogeny Reconstructions

Identified MADS-domain sequences were aligned with known MADS domains of *A. thaliana*, *O. sativa*, *P. trichocarpa, C. sativus, G. max*, and *V. vinifera* using MAFFT [33]. An unrooted Maximum Likelihood tree was constructed using RAxML [34]. The different types of MADS-domain proteins were identified from the phylogeny based on the position of the characterized proteins of *A. thaliana*, *P. trichocarpa*, and *O. sativa* [8,9,21]. Separate phylogenies were then reconstructed for Type I and Type II genes, again using MAFFT for alignments and RAxML to obtain Maximum Likelihood phylogenies.

## 3. Results and Discussion

### 3.1. Identified MADS-Box Genes

We tested our pipeline using the genomes of *A. thaliana* and *O. sativa*. All but one and four of the known MADS-box genes of *A. thaliana* and *O. sativa*, respectively, were retrieved from their predicted CDS. In case of *A. thaliana*, five additional potential MADS-box genes have then been identified from the genome while, for *O. sativa*, one additional gene was detected.

From the 17 plant genomes we newly identified 2,060 MADS-box genes, of which 1,057 were classified as Type I and 990 were classified as Type II (Table 2; all identified as well as previously known MADS-box genes used in this study are available as Supplementary Data 2–4). The largest number of MADS-box genes was identified in *S. tuberosum* (265) of which 173 genes belong to Type I. This is the second highest number of Type I genes found in all species. The highest number of Type I genes was found in *C. papaya* where 229 of 262 MADS-box genes are Type I. Papaya was shown to have a comparably low number of genes, so the high number of MADS-box genes is surprising [35]. The highest number of Type II genes was identified in *M. domestica* with 113 Type II MADS-box genes. *R. communis* has the lowest number of MADS-box genes in the flowering plants studied at 60 genes. However, this number is still higher than the number of MADS-box genes previously identified in *C. sativus* with 50 genes [24]. Low numbers of Type I genes in flowering plants were observed in *C. sativus* (11 genes) and *Z. mays* (14 genes). The lowest number of Type II genes in a flowering plant was found in *M. truncatula* with 24 genes. Thus, the range of MADS-box genes in flowering plants is greater for Type I genes (11 to 229 genes) than for Type II genes (24 to 113 genes).

### 3.2. Phylogenies of MADS-Box Genes

**Figure 4:**
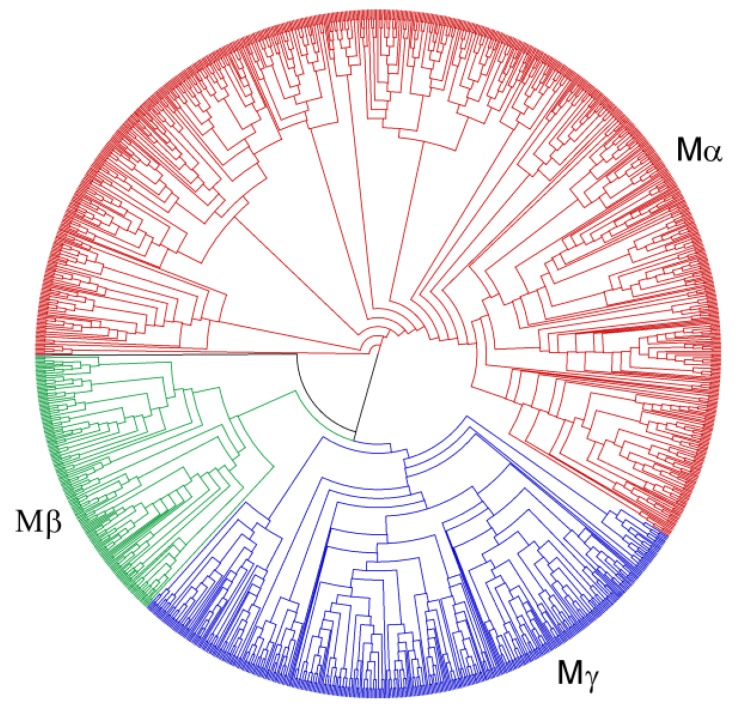
Phylogeny of Type I MADS-box genes from 24 plant species including known genes from *A. thaliana*, *P. trichocarpa*, *G. max*, *C. sativus*, *O. sativa*, *S. moellendorffii*, and *P. patens* and newly annotated genes. Groups of Type I genes are indicated by different colours and their names are shown. Black indicates genes that could not be assigned unambiguously to one of the groups.

**Figure 5:**
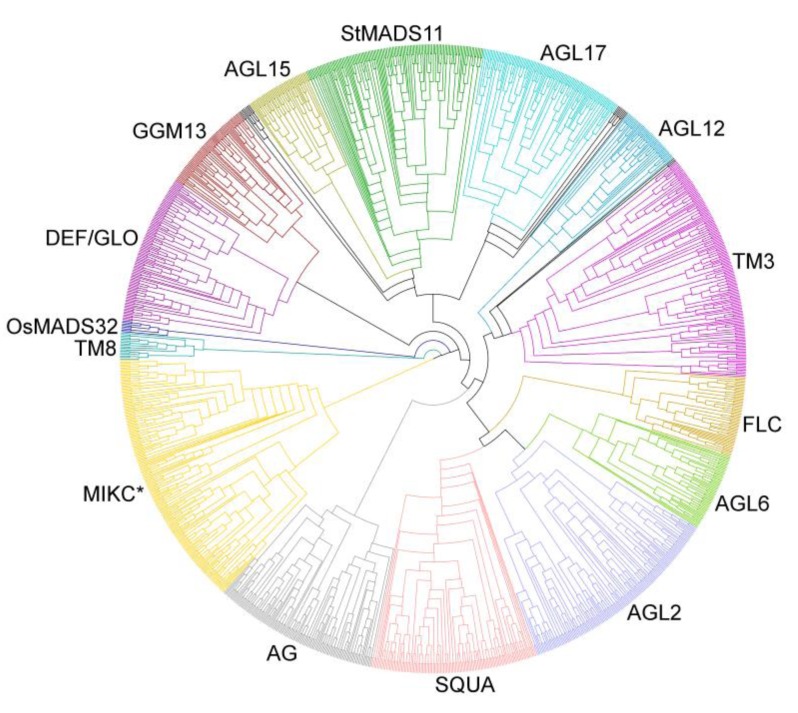
Phylogeny of Type II MADS-box genes from 24 plant species including known genes from *A. thaliana*, *P. trichocarpa*, *G. max*, *C. sativus*, *O. sativa*, *S. moellendorffii*, and *P. patens* and newly annotated genes. Groups of Type II genes are indicated by different colours and their names are shown. Black indicates genes that could not be assigned unambiguously to one of the groups.

We have reconstructed separate maximum likelihood phylogenies of the Type I and Type II MADS-box genes (Figure 4, Figure 5; Supplementary Figures 1 and 2). The newly identified genes were assigned to the different groups of MADS-box genes according to their position in the phylogenies relative to the previously classified genes of *A. thaliana* and *O. sativa* [8,9]. Unlike a previous classification, OsMADS90 and OsMADS91 fall into the Mα group rather than into the Mβ group in our phylogeny. Furthermore, OsMADS86 is adjacent to the Mβ and Mγ groups rather than in the Mγ group in our phylogeny. Apart from this, the three groups may well be monophyletic.

**Table 2:** Total number of MADS-box genes in 24 plant species and classification into Type I and Type II genes. The complete set of MADS-box genes was previously known for *P. trichocarpa*, *G. max*, *C. sativus*, *A. thaliana*, *O. sativa*, *S. moellendorffii*, and *P. patens* (indicated by bold writing) [6,8,9,21,22,23,24]. * The remaining MADS-box genes could not be classified into Type I or Type II.

Species [Genome reference]	Total	Type I	Type II
*Manihot esculenta* [36]	85	28	57
*Ricinus communis* [37]	60	23	37
*Linum usitatissimum* [38]	123	45	78
***Populus trichocarpa* [39]**	**95**	**35**	**60**
*Medicago truncatula* [40]	81	57	24
Glycine max* [41]	**180**	**82**	**96**
***Cucumis sativus*** [42]	**50**	**11**	**39**
*Prunus persica* [43]	85	41	44
*Malus domestica* [44]	179	66	113
***Arabidopsis thaliana* [45]**	**105**	**59**	**46**
*Arabidopsis lyrata* [46]	110	61	49
Carica papaya * [35]	262	229	30
Citrus sinensis * [47]	89	24	64
Vitis vinifera * [48]	90	44	44
Solanum lycopersicum * [49]	190	127	62
Solanum tuberosum * [50]	265	173	86
*Sorghum bicolor* [51]	67	24	43
*Zea mays* [52]	69	14	55
*Setaria italica* [53]	137	41	96
***Oryza sativa* [54]**	**71**	**29**	**42**
*Brachypodium distachyon* [55]	75	35	40
*Musa accuminata* [56]	93	25	68
***Selaginella moellendorffii* [57]**	**19**	**13**	**6**
***Physcomitrella patens* [58]**	**23**	**7**	**16**
Total*	2,603	1,293	1,295

In the phylogeny of Type II genes, the group of MIKC* genes and the different groups of MIKC^C^‑group genes are well identifiable (Figure 5; Supplementary Figure 2). All of the previously defined groups may well be monophyletic also in our phylogeny. All of the groups contain a similar number of genes from nearly all species analyzed indicating that Type II MADS-box genes are rarely duplicated or lost or are about as frequently duplicated as lost after the establishment of the different groups.

Type I genes have been recognized to have faster birth-and-death rates than Type II genes before, when MADS-box genes in *A. thaliana* and *O. sativa* were studied [28]. The lineages that led to *A. thaliana* and *O. sativa* diverged around 150 million years ago [59]. Here, we show that, even when examining a larger number and more closely related species, still a large number of lineage-specific expansions can be seen for Type I genes. For example, when comparing the number of independent duplications in *A. thaliana* which are not found in *A. lyrata* and *vice versa* (Table 3), it becomes apparent that even within the last five million years after the divergence of these two *Arabidopsis* species [60], many duplications happened for Type I but not for Type II MADS-box genes.

**Table 3:** Number of independent duplications of Type I and Type II MADS-box genes in *A. thaliana* as compared to *A. lyrata.*

Species	Type I	Type II
*A. thaliana*	6	0
*A. lyrata*	9	2

### 3.3. Different Evolutionary Patterns of Type I and Type II MADS-Box Genes

Comparing the phylogenies of Type I and Type II MADS-box genes, it becomes apparent that there are more large species-specific groups of Type I genes than of Type II genes (Table 4). While there are 10 groups containing 10 or more genes from the same species for Type I genes, only three such groups exist for Type II genes. Also, the number of groups containing five to nine genes from the same species is larger for Type I genes at 27 groups as compared to eight groups for Type II genes. This bias is not due to a bias in gene number as the number of Type I (1,293) and Type II genes (1,295) is nearly identical in the 24 flowering plant species analyzed. Most prominent are expansions of Type I genes for *C. papaya*. There is one group comprised of 36 genes and another group of 58 genes from *C. papaya*. Why these genes have undergone such massive expansions in papaya in particular, is unknown. We did not include gymnosperm genomes in this study. However, the genome of *Picea abies* reveals that the situation might be different in gymnosperms where Type I MADS-box genes might be rare [61].

**Table 4:** Number of species-specific groups of a certain size in our dataset for Type I and Type II genes.

Size	Type I	Type II
≥10	10	3
9	1	0
8	5	2
7	4	1
6	8	2
5	9	3

Which mechanisms lead to the higher rate of duplications or the higher rate of ‘short-term’ retention of Type I genes as compared to Type II genes, and to the different evolutionary dynamics of both gene types in general, is largely unknown. 

According to the gene balance hypothesis genes that are more connected, e.g., due to protein-protein interactions of their gene products, are more likely retained after whole genome or large-scale duplications than after tandem or other small-scale duplications [62,63]. Type II MADS-domain proteins are probably involved in a higher number of complexes and in more complex multimers than Type I proteins [64,65], possibly due to the presence of the K domain that facilitates protein-protein interactions [66]. Therefore, Type II genes may have higher probabilities than Type I genes to be maintained after whole genome duplications and to be lost after small-scale duplications due to purifying selection that tries to keep genes in balance. Thus, the gene balance hypothesis might at least partially explain the differences in evolutionary patterns of Type I and Type II genes.

Secondly, there may be positive selection on the duplication and retention of certain genes after small scale duplications. This has been hypothesized for genes which are involved in coevolutionary scenarios, e.g., in some reproductive processes [67]. Compared to Type II genes Type I genes are less well studied, but the few Type I genes for which functions are known are involved in female gametophyte, embryo, and seed development and hence quite directly in reproduction [7,68]. There is evidence that some Type I genes in *A. thaliana* are involved in the development of interspecific barriers and hence may promote speciation [69]. Thus, the difference in the evolutionary patterns between Type I and Type II genes may also be explained by selection for diversity and neofunctionalization of Type I genes.

However, in addition to selection regimes also mutation patterns could be different for Type I and Type II genes. It has been hypothesized that Type I MADS-box genes have been highjacked by transposons, or have an intrinsic transposon activity [70], and, thus, might duplicate more often than ‘ordinary’ genes including Type II MADS-box genes. Furthermore, Type I genes are shorter and have less exons than Type II genes. Hence, the chance of Type I genes being duplicated as functional units might be higher than that of the larger Type II genes.

To find out more about the mutational mechanisms by which Type I genes are duplicated, we investigated the chromosomal position of the Type I genes with lineage-specific duplications in *A. thaliana* and *A. lyrata*. We found two tandem, three proximal, and ten distal duplications (as defined in [71]). The distal duplications might be caused by transposon activity. Hence, the investigated Type I duplications indicate both easy duplicatability and dispersal by transposon activity. Further studies are needed to gain more insights into the reasons for the different evolutionary rates of Type I and Type II genes.

## 4. Conclusions

Here we have identified the complete set of MADS-box genes in 17 plant species (some minor refinements in the future notwithstanding). We have analyzed more than 2,600 MADS-box genes and classified them into the known types. Our results corroborate a previous finding that Type I genes have a faster evolutionary rate than Type II genes and show that the number of duplications of Type I genes is high even in short time frames as can be recognized from the comparison of the closely related species *A. thaliana* and *A. lyrata*. Our study provides the basis for more elaborate studies on the striking differences in the evolutionary patterns of Type I and Type II genes and on the function of MADS-box genes in different plant species.

## Supplementary Material

**Figure 1.** Phylogeny of Type I MADS-box genes from 24 plant species.

**Figure 2.** Phylogeny of Type II MADS-box genes from 24 plant species.

**Data 1.** pipeline.zip, Perl scripts to predict MADS-box genes in sequences plant genomes.

**Data 2.** TypeI.fa, Type I MADS-box genes in angiosperms as identified before and in this study.

**Data 3.** TypeII.fa, Type II MADS-box genes in angiosperms as identified before and in this study.

**Data 4.** unassigned.fa, MADS-box genes in angiosperms identified in this study which could not be assigned to Type I or Type II unambiguously.

Supplementary File 1 Supplementary (ZIP, 8248 KB)
