# Supplementary material for: Phylogenomics of MADS-Box Genes in Plants — Two Opposing Life Styles in One Gene Family
Source: Biology (Basel). 2013 Sep 12;2(3):1150–64. doi: 10.3390/biology2031150 (PMC3960868; doi:10.3390/biology2031150)
Supplement: Supplementary File 1 — Supplementary (ZIP, 8248 KB) [file biology-02-01150-s001.zip › supplementary/SupplementaryMaterial_Legends.docx]

Supplementary Material – Legends

**Figure 1.** Phylogeny of Type I MADS-box genes from 24 plant species including known genes from *A. thaliana*, *P. trichocarpa*, *G. max*, *C. sativus*, *O. sativa*, *S. moellendorffii* and *P. patens* and newly annotated genes. Groups of Type I genes are indicated by different colours as shown in Figure 4 of the manuscript. Lineage-specific expansions comprising five to ten genes are indicated by blue colouring of the genes involved. Lineage-specific expansions comprising more than ten genes are indicated by bold edges. Species abbreviations are as follows Me, *Manihot esculenta*; Rc. *Ricinus communis*; Lu, *Linum usitatissimum*; Pt, *Populus trichocarpa*; Mt, *Medicago truncatula*; Gm, *Glycine max*; Cs, *Cucumis sativus*; Ppe, *Prunus persica*; Md, *Malus domestica*; AT, *Arabidopsis thaliana*; Al, *Arabidopsis lyrata*; Cp, *Carica papaya*; Csi, *Citrus sinensis*; Vv, *Vitis vinifera*; Sl, *Solanum lycopersicum*; St, *Solanum tuberosum*; Sb, *Sorghum bicolor*; Zm, *Zea mays*; Si, *Setaria italica*; Os, *Oryza sativa*; Bd, *Brachypodium distachyon*; Ma, *Musa accuminata*; Sm, *Selaginella moellendorffii*; Pp, *Physcomitrella patens*. All MADS-box genes identified from a species were numbered and where applicable, the name by which a gene was previously labeled was used (additionally), the identifier of the predicted coding sequence as provided by the genome database was added to the name or the chromosome/scaffold number where the gene was identified was added. The numbers on the nodes are bootstrap values for 100 replicates.

**Figure 2.** Phylogeny of Type II MADS-box genes from 24 plant species including known genes from *A. thaliana*, *P. trichocarpa*, *G. max*, *C. sativus*, *O. sativa*, *S. moellendorffii* and *P. patens* and newly annotated genes. Groups of Type II genes are indicated by different colours as shown in Figure 5 of the manuscript. Labelling of lineage-specific expansions, species abbreviations, naming of the genes and numbers are as described for Supplementary Figure 1.

**Data 1.** pipeline.zip, Contains perl-scripts, customized Hidden Markov Model (MADS_short2.hmm) together with an instruction (README.txt) to use our pipeline to predict MADS-box genes in sequences plant genomes.

**Data 2.** TypeI.fa, Type I MADS-box genes in angiosperms as identified before and in this study. Species abbreviations and naming of the genes are as described for Supplementary Figure 1.

**Data 3.** TypeII.fa, Type II MADS-box genes in angiosperms as identified before and in this study. Species abbreviations and naming of the genes are as described for Supplementary Figure 1.

**Data 4.** unassigned.fa, MADS-box genes in angiosperms identified in this study which could not be assigned to Type I or Type II unambigously. Species abbreviations and naming of the genes are as described for Supplementary Figure 1.
